# Comparable efficacy of extraoral and intraoral photobiomodulation in preventing oral mucositis during high-dose methotrexate therapy in pediatric oncology: A single-blind randomized controlled trial

**DOI:** 10.1007/s00520-026-11372-9

**Published:** 2026-10-19

**Authors:** Isabela de Oliveira Plugge Freitas, Isabela Schuartz, Giovanna Leal Klein Renz, Rafael Zancan Mobile, Mariana Lena Sassi França, Mara Albonei Dudeque Pianovski, Laurindo Moacir Sassi, Melissa Rodrigues de Araujo

**Affiliations:** 1 Department of Stomatology, Federal University of Paraná (UFPR), Curitiba, Paraná Brazil; 2 Hospital Erasto Gaertner, Curitiba, Paraná Brazil; 3 National Institute of Science and Technology in Translational Biophotonics in Dentistry, INCT-BIOFOTO BUCAL, Porto Alegre, Brazil

**Keywords:** Low-level light therapy, Oral mucositis, Pediatrics, Oral medicine, Methotrexate, Medical oncology

## Abstract

**Purpose:**

This prospective randomized controlled clinical study compared intraoral (IO) and extraoral (EO) photobiomodulation (PBM) for the prevention of chemotherapy-induced oral mucositis (OM) in pediatric oncology patients.

**Methods:**

A total of 155 patients (1–18 years), receiving high-dose methotrexate (HDMTX) for leukemia, lymphoma, or osteosarcoma, were randomized to PBM-IO (*n* = 73) or PBM-EO (*n* = 82). Preventive PBM was administered daily from hospital admission through discharge. Both protocols used red light with equivalent dosimetry (660 nm, 100 mW, 1 J per point). PBM-EO required 30 s/session, whereas PBM-IO required approximately 6 min, excluding patient handling time. OM occurrence, severity (WHO scale), plaque index, and pain (numeric rating scale) were assessed on days 0, 3, 5, and 10 after HDMTX infusion.

**Results:**

The overall OM occurrence was 35.5%, with no significant difference between PBM-IO (41.1%) and PBM-EO (30.5%) groups (*p* = 0.168). No statistically significant differences were observed between groups for OM severity, pain, salivary pH, or plaque index at any time point (*p* > 0.05). Multivariable ordinal logistic regression demonstrated that PBM modality was not independently associated with OM severity. As expected, increasing age (OR = 1.14; 95% CI: 1.04–1.25; *p* = 0.004) and higher HDMTX dose (OR = 1.24; 95% CI: 1.03–1.49; *p* = 0.021) were significantly associated with greater OM severity. Median pain scores remained 0 at all assessment points in both groups.

**Conclusion:**

Extraoral PBM demonstrated comparable effectiveness to intraoral for OM prevention in pediatric patients undergoing HDMTX chemotherapy, while reducing application time.

## Introduction

Oral mucositis (OM) is the most frequent and debilitating oral side effect associated with chemotherapy, characterized by erythema, erosion, and ulceration of the oral mucosa [1, 2]. The incidence of OM is especially high in pediatric patients, particularly those receiving intensive chemotherapy protocols, such as high-dose methotrexate (HDMTX), which targets tissues with high cellular turnover, including the oral mucosa [1–4]. Severe OM is associated with intense pain, increased risk of systemic infection, nutritional impairment, and prolonged hospitalization, often leading to dose reductions or interruptions in cancer therapy [3, 5–7].

Given its clinical impact, effective preventive strategies for OM are essential [2, 3]. Photobiomodulation (PBM) has been recommended by the Multinational Association of Supportive Care in Cancer and the International Society of Oral Oncology (MASCC/ISOO) for the prevention and management of OM [8, 9]. In pediatric populations specifically, growing evidence supports the efficacy of PBM in preventing chemotherapy-induced OM [38, 39].

Traditionally, PBM is delivered through intraoral (IO) application, which requires direct access to mucosal tissues. However, this approach may be challenging in pediatric patients due to discomfort, limited cooperation, and the presence of extensive or painful lesions. In this context, extraoral (EO) or transcutaneous PBM has emerged as a potential alternative [10], allowing non-invasive application and improved patient tolerance while effectively reaching oropharyngeal regions involved in the pathogenesis of OM [11–13].

Despite these practical advantages, the clinical effectiveness of PBM-EO compared with the traditional IO approach remains poorly established, particularly in pediatric oncology populations. We hypothesized that extraoral photobiomodulation would provide preventive clinical outcomes comparable to those achieved with intraoral photobiomodulation. Therefore, this study aimed to compare the clinical efficacy of extraoral and intraoral PBM for the prevention of oral mucositis in pediatric patients undergoing high-dose methotrexate chemotherapy in a specialized care setting.

## Materials and methods

This was a prospective, randomized, controlled clinical study conducted at a reference pediatric oncology hospital. The study was approved by the Institutional Research Ethics Committee (CAAE: 52,808,121.1.0000.0098) and conducted in accordance with the Declaration of Helsinki.

Sample size calculation was based on the expected occurrence of oral mucositis (WHO ≥ grade 1) as the primary outcome, an expected incidence of 10% was considered, based on data reported for a comparable pediatric population by Guimaraes et al. [14]. With an 18-percentage-point increase, established as the minimum clinically relevant difference between PBM modalities and considering a two-sided α error of 5% and a statistical power of 80%, the estimated sample size was 71 participants per group (142 total).

### Participants

The sample consisted of pediatric patients (1–18 years) of both sexes undergoing chemotherapy for malignant neoplasms (leukemia, lymphoma, and osteosarcoma) involving high-dose methotrexate, administered alone or in combination with the MADIT protocol (methotrexate, cytarabine, and dexamethasone).

Inclusion criteria were patients receiving HDMTX-based chemotherapy at any stage of treatment, able to attend all scheduled evaluations, and whose parents or legal guardians provided written informed consent.

Exclusion criteria from the study included active oral infections, OM resulting from a previous chemotherapy cycle at baseline, and use of chemotherapeutic regimens outside the study protocol.

After randomization, participants without any follow-up assessment after D3 (who did not attend either the D5 or the D10 assessment) were excluded from the analysis, as their follow-up was insufficient to assess the primary outcome.

Eligible patients were screened and evaluated before chemotherapy initiation. After consent, participants were randomly allocated (1:1) to intraoral PBM (PBM-IO) or extraoral PBM (PBM-EO) using a computer-generated randomization sequence (random.org). Allocation was applied sequentially as participants were enrolled.

All patients and their parents/caregivers received standardized oral hygiene instructions and information regarding potential oral complications from the research team before treatment initiation. Instructions included gentle toothbrushing with a soft-bristled toothbrush and fluoride toothpaste three times a day and flossing at least once a day. No mouthwashes were prescribed, as they were not part of the institutional routine. The same oral care was provided to both groups.

Due to the nature of the interventions, blinding of patients and operators was not feasible. However, outcome assessment was performed by an examiner who was blinded to group allocation, characterizing this as a single-blind study.

### PBM-IO group

Patients received PBM using a diode laser device (Therapy EC®, DMC, São Carlos, Brazil). Treatment started one day before chemotherapy and was performed daily during hospitalization (3–5 days).

The device consisted of an AlGaInP diode laser (660 nm, 100 mW). Each point received 1 J over 10 s (energy density: 10 J/cm^2^; beam area: 0.1 cm^2^). The laser was applied in direct contact with the oral mucosa.

**Fig. 1:**
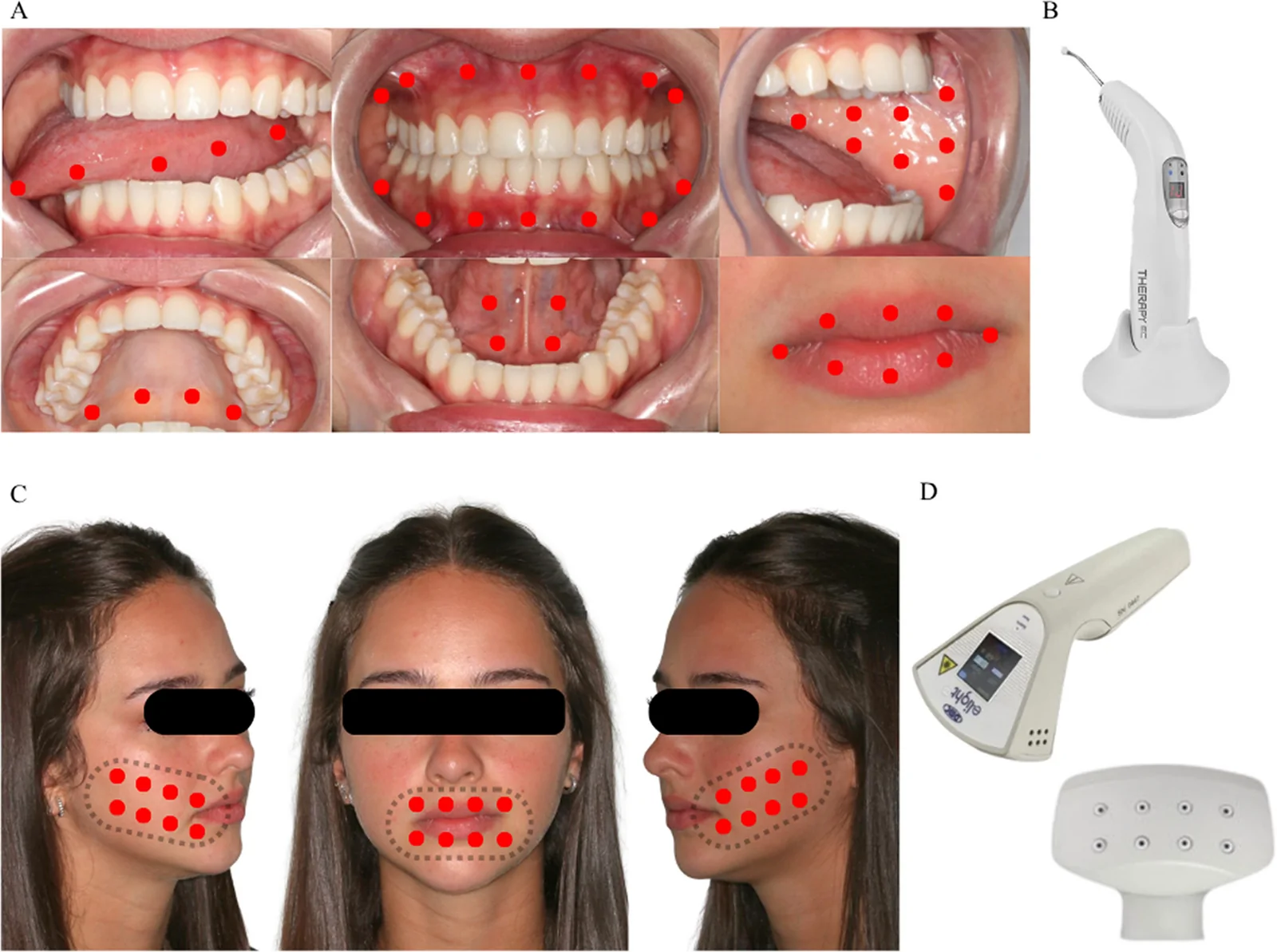
Schematic representation of intraoral and extraoral PBM application points, (**A**) PBM-IO application points; (**B**) PBM-IO laser device; (**C**) PBM-EO application points; (**D**) PBM-EO laser device. Images published with the model’s authorization. Photographs and device images showing intraoral and extraoral photobiomodulation applicationpoints, marked as red dots, and the two laser devices used

Application sites included buccal mucosa, labial mucosa, floor of the mouth, tongue borders, tongue tip, soft palate, and labial commissures, according to a previously described protocol [15]. Application sites are illustrated in Fig. 1. The effective irradiation time per session was approximately 6 min, excluding patient handling time.

### PBM-EO group

Patients received PBM using a cluster diode laser device (E-light RL®, DMC, São Carlos, Brazil) composed of eight AlGaInP emitters (660 nm, 100 mW). Each point was irradiated for 10 s, delivering 1 J. Energy density varied according to emitter position (4 central: 34.5 J/cm^2^; 4 lateral: 4.3 J/cm^2^). Treatment started one day before chemotherapy and was performed daily during hospitalization (3–5 days).

Applications were performed in direct contact with the skin at three extraoral points: the bilateral buccinator regions and the orbicularis oris muscle (Fig. 1). The effective irradiation time per session was 30 s, excluding patient handling time.

### Procedures common to both groups

All PBM procedures were performed by a team of two dentists and five final-year dental students, previously trained. Oral mucositis assessments were performed by a trained and experienced examiner, who was blinded to group allocation (PBM-IO and -EO).

Patients who developed oral mucositis during follow-up received additional PBM sessions as therapeutic management. In these cases, an intraoral PBM protocol was applied regardless of initial group allocation. This intervention was defined a priori as a rescue therapy, aiming to ensure ethical management of symptomatic patients. For analytical purposes of outcomes, participants were maintained in their originally assigned groups, following an as-randomized analysis approach.

### Infection control and safety

Strict infection control protocols were implemented for all PBM sessions (both PBM-IO and PBM-EO). Prior to each session, the laser tip was disinfected with 70% alcohol and individually wrapped in a disposable PVC plastic barrier film to prevent cross-contamination. Following each procedure, the barrier film was removed, and the laser tip was disinfected again. All individuals present in the room during application (including the operator, patient, and accompanying parents/guardians) wore appropriate wavelength-specific protective eyewear. Protective glasses were thoroughly washed with water and neutral soap after each use.

### Primary outcome

The primary outcome was the incidence and severity of oral mucositis.

Data were recorded using a standardized electronic form, including demographic and clinical variables. Patients were evaluated at four time points: D0 (one day before chemotherapy initiation), D3 (third day after chemotherapy initiation), D5 (fifth day), and D10 (tenth day). The unit of analysis was the individual patient.

Oral mucositis was assessed according to the World Health Organization (WHO) Oral Toxicity Scale, classified as follows: grade 0 (no alterations), grade I (erythema), grade II (erythema and ulcers with solid diet), grade III (ulcers requiring liquid diet), and grade IV (ulcers with inability to tolerate oral feeding) [16]. Severe oral mucositis was defined as WHO Grade > 2 (Grades 3 and 4). To ensure consistency and reproducibility, all oral examinations and mucositis scorings were performed by the same trained clinical researcher throughout the entire study period.

### Secondary outcomes

Secondary outcomes included: Pain intensity; Oral hygiene status; Salivary pH.

Pain was assessed in all patients at each time point. Most children were able to self-report, as pediatric oncology patients are routinely asked to rate their pain during treatment; for very young children unable to self-report, pain was reported by parents or caregivers using the same scale. Pain intensity was assessed using an eleven-point Numeric Rating Scale (NRS-11), ranging from 0 (no pain) to 10 (worst possible pain) [17].

Oral hygiene status was assessed using a simplified visible plaque index. A dental probe was gently passed along the gingival margin to detect the presence of supragingival plaque. Plaque accumulation was recorded dichotomously as present or absent, based on visual and tactile assessment.

Unstimulated saliva was collected using a cotton roll placed intraorally for approximately one minute without mechanical stimulation. After collection, samples were kept under cooled conditions and subsequently stored frozen at − 20 °C until analysis. Before pH evaluation, samples were thawed in a laboratory oven and centrifuged to obtain the supernatant. Salivary pH was then measured using a calibrated digital pH meter (Phtek®—PHS 3B), with calibration performed using standard buffer solutions prior to analysis.

Salivary pH was measured at the same four time points as oral mucositis (D0, D3, D5 and D10), and pain intensity was assessed at D3, D5 and D10. The visible plaque index was assessed only at baseline (D0) to characterize oral hygiene status before chemotherapy.

### Statistical analysis

Statistical analyses were performed using Jamovi software (version 2.6.44). The normality of continuous variables was evaluated using the Shapiro–Wilk test. All continuous variables exhibited a non-normal distribution (*p* < 0.05). Categorical variables were summarized as absolute and relative frequencies, while continuous variables were described as medians and interquartile ranges (IQR), due to the non-normal distribution of the data. Comparisons between groups (PBM-IO vs. PBM-EO) were performed using the chi-square test for categorical variables and the Mann–Whitney *U* test for continuous or ordinal variables. An ordinal logistic regression model was used to assess the association between PBM modality and mucositis severity at D10, as this was the only evaluation time point in which severe oral mucositis cases were observed.

Overall oral mucositis occurrence (yes/no) was compared between groups using the Pearson Chi-Square test. Additionally, to account for the ordinal nature of oral mucositis severity grades, comparisons of severity frequencies between groups at each time point were conducted using Kendall’s tau-b test. Multicollinearity among covariates (age and HDMTX dose) was evaluated using the Variance Inflation Factor (VIF) and Tolerance metrics. Values of VIF < 5.0 and Tolerance > 0.20 were considered indicative of no significant multicollinearity. The significance level was set at 5% (*p* < 0.05). Results were expressed as odds ratios (OR) and 95% confidence intervals.

## Results

**Fig. 2:**
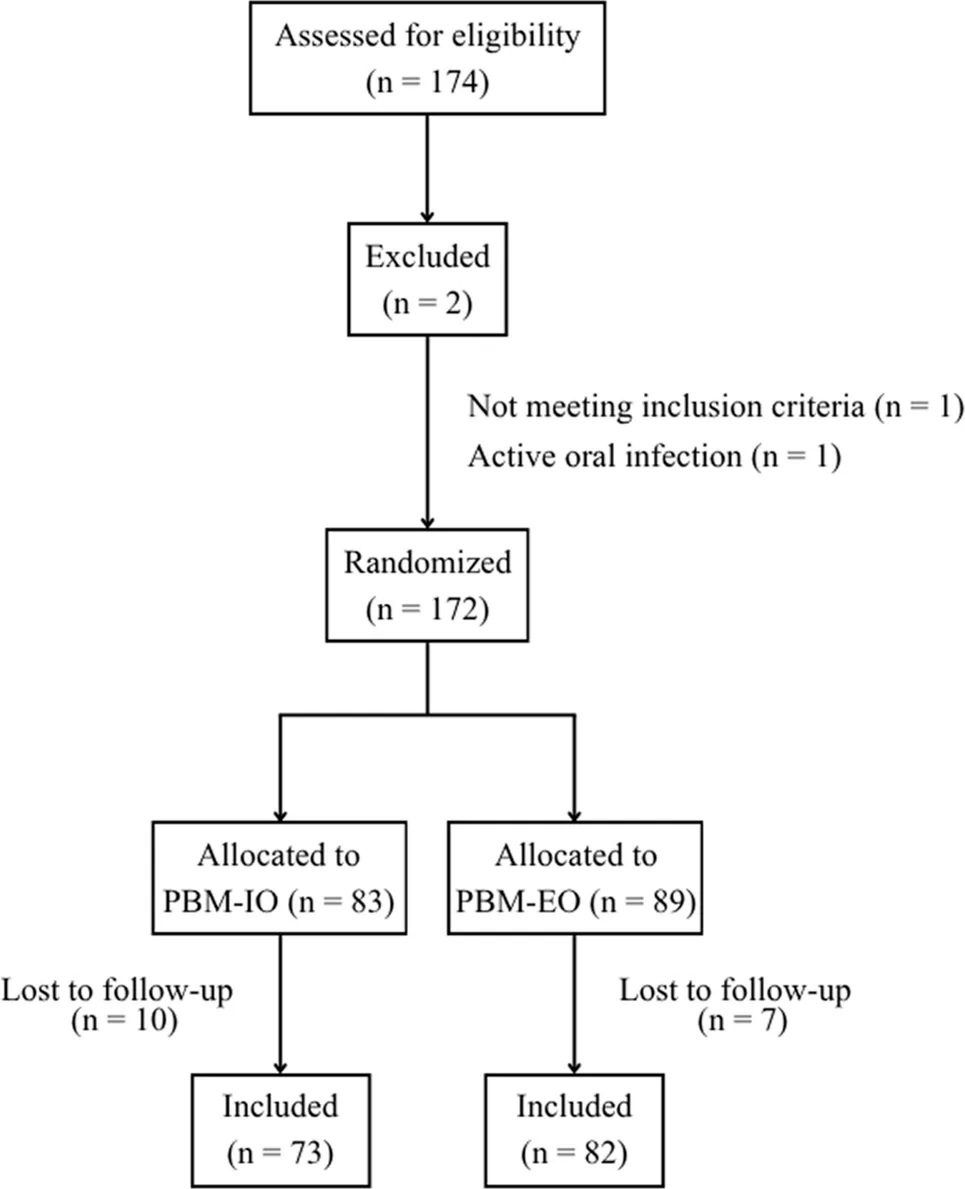
Flow diagram of patient selection, allocation, follow-up, and analysis. Flow diagram of the trial: 174 patients assessed, 172 randomized, and 155 included in the analysis (73 intraoral, 82 extraoral)

The study was conducted between July 2022 and July 2025. A total of 174 patients were assessed for eligibility, of whom 172 were randomized. Seventeen participants (IO: *n* = 10; EO: *n* = 7) did not return to the hospital for the D5 and D10 assessments after discharge and were excluded from the analysis, resulting in a final sample of 155 patients (Fig. 2). Of these, 73 patients (47.1%) were allocated to the PBM-IO group and 82 patients (52.9%) to the PBM-EO group.

The baseline characteristics of the study population are presented in Table 1. A statistically significant difference was observed between groups regarding age (*p* = 0.004; Mann–Whitney *U* test), with the PBM-IO group showing a higher median age (8 years [IQR: 6–12]) compared to the PBM-EO group (6 years [IQR: 3–10]).

**Table 1:** Baseline demographic and clinical characteristics of pediatric oncology patients undergoing HDMTX according to PBM modality (PBM-IO vs. PBM-EO)

Variable		PBM-IO	PBM-EO	Total	*p* value
Age (years)	Median (IQR)	8 (6–12)	6 (3–10)	-	*p* = 0.004
Sex	Female	22 (30.1%)	28 (34.1%)	50 (32.3%)	*p* = 0.594
	Male	51 (69.9%)	54 (65.9%)	105 (67.7%)	
Diagnosis	Acute Lymphoblastic Leukemia (ALL)	53 (72.6%)	60 (73.2%)	113 (72.9%)	*p* = 0.616
	Osteosarcoma	13 (17.8%)	11 (13.4%)	24 (15.5%)	
	Non-Hodgkin Lymphoma	7 (9.6%)	11 (13.4%)	18 (11.6%)	
HDMTX dose (g/m^2^) ᵃ	Median (IQR)	5 (2.5–5)	5 (2.5–5)	-	*p* = 0.392
Visible Plaque Index	Present	45 (61.6%)	45 (54.9%)	90 (58.1%)	*p* = 0.394
	Absent	28 (38.4%)	37 (45.1%)	65 (41.9%)	

^a^Data available for 80 patients (PBM-IO: *n* = 36; PBM-EO: *n* = 44)

The sample was predominantly male (67.7%), with no significant difference in sex distribution between groups (*p* = 0.594). Regarding clinical characteristics, most patients were diagnosed with acute lymphoblastic leukemia (72.9%), followed by osteosarcoma and non-Hodgkin lymphoma, with no significant differences between groups (*p* = 0.616). Oral hygiene status at baseline, assessed by the presence of visible dental plaque, was also comparable between groups (*p* = 0.394). PBM duration was comparable between groups, with a median of 4 days (IQR: 4–5) in both groups.

**Fig. 3:**
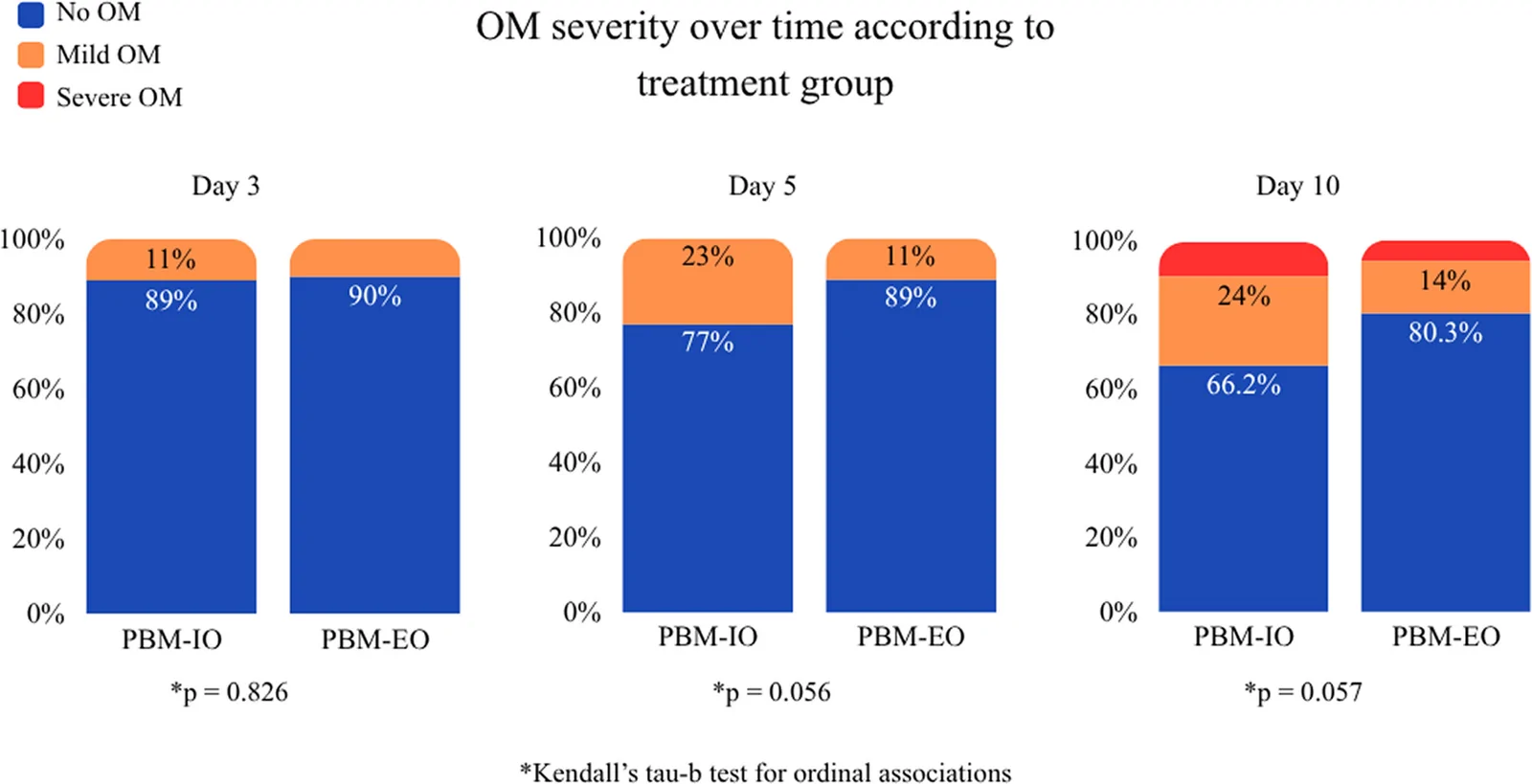
Overall oral mucositis severity over time according to treatment group. Stacked bar charts comparing oral mucositis severity in the intraoral and extraoral groups on days 3, 5 and 10; most patients had no mucositis, and differences were not significant

The overall occurrence of oral mucositis in the study population was 35.5%. No statistically significant difference was observed between groups, with OM occurring in 41.1% of patients in the PBM-IO group and 30.5% in the PBM-EO group (χ^2^ = 1.90; *p* = 0.168) (Table 2, Fig. 3).

**Table 2:** Oral Mucositis occurrence, pain and salivary pH

	PBM-IO		PBM-EO		Total		*p*-value
	N	Column %	N	Column %	N	Column %	
Overall OM occurrence							
Yes	30	41.1%	25	30.5%	55	35.5%	*p = 0.168
No	43	58.9%	57	69.5%	100	64.5%	
Pain (NRS-11)							
Day 3	73	0 (0–0)	80	0 (0–0)	153	-	**p = 0.447
Day 5	70	0 (0–1.75)	73	0 (0–0)	143	-	**p = 0.221
Day 10	64	0 (0–0)	72	0 (0–0)	136	-	**p = 0.187
Pain in patients with OM							
Day 3	30	0 (0–0)	24	0 (0–0)	54	-	**p = 0.839
Day 5	28	3 (0–4.25)	21	1 (0–4)	49	-	**p = 0.531
Day 10	28	1 (0–3.25)	23	0 (0–3.5)	51	-	**p = 0.853
Salivary pH							
Day 0	33	7.94 (7.64–8.26)	38	7.67 (7.08–8.34)	71	-	**p = 0.249
Day 3	30	7.88 (7.18–8.49)	35	8.06 (7.44–8.40)	65	-	**p = 0.708
Day 5	26	7.91 (7.25–8.43)	29	7.98 (7.60–8.33)	55	-	**p = 0.781
Day 10	26	7.89 (7.39–8.28)	24	7.73 (7.22–8.18)	50	-	**p = 0.607

Data are presented as *n* (%) for categorical variables and median (IQR) for continuous variables

*Pearson Chi-Square test

**Mann–Whitney *U* test

Additionally, unadjusted analyses showed no statistically significant differences between groups regarding OM severity at days 3 (*p* = 0.826), 5 (*p* = 0.056), and 10 (*p* = 0.057) (Table 2).

Separate multivariable ordinal logistic regression models were performed to assess the association between PBM modality and OM severity at day 10, adjusting for age and HDMTX dose in separate models because HDMTX dose data were available for only 80 patients, of whom 61 had a D10 assessment. Multicollinearity diagnostics indicated no significant collinearity between age and HDMTX dose (VIF = 1.34; tolerance = 0.75). The full results of both models are presented in Table 3. As expected, increasing age and higher HDMTX dose were significantly associated with greater OM severity (Table 3). Consistently, among patients with available dose data (*n* = 80), those who developed OM received higher HDMTX doses than those who did not (*p* < 0.001; Mann–Whitney *U* test).

**Table 3:** Multivariable ordinal logistic regression models for OM severity at day 10

	OR	95% CI	*p* value
Model 1: Adjusted for age (*N* = 136)			
PBM-EO vs. PBM-IO (ref.)	0.57	0.25–1.25	0.164
Age (years)	1.14	1.04–1.25	**0.004**
Model 2: Adjusted for HDMTX dose (*N* = 61)			
PBM-EO vs. PBM-IO (ref.)	0.36	0.09–1.51	0.163
HDMTX dose (g/m^2^)	1.24	1.03–1.49	**0.021**

*OR*, Odds Ratio; *CI*, Confidence Interval; *PBM-EO*, Extraoral Photobiomodulation; *PBM-IO*, Intraoral Photobiomodulation; *HDMTX*, High-Dose Methotrexate. Note: Model 2 was conducted on a reduced subsample due to the availability of complete HDMTX dosage records. Multicollinearity diagnostics: age and HDMTX dose (VIF = 1.34; Tolerance = 0.75)

Regarding patient-reported pain, scores were consistently low in both groups, with a median of 0 at all time points. No statistically significant differences were observed between PBM-IO and PBM-EO at day 3 (median 0 [IQR: 0–0] in both groups; *p* = 0.447), day 5 (PBM-IO: median 0 [IQR: 0–1.75]; PBM-EO: median 0 [IQR: 0–0]; *p* = 0.221), or day 10 (median 0 [IQR: 0–0] in both groups; *p* = 0.187; Mann–Whitney *U* test). Among patients who developed OM (PBM-IO: *n* = 30; PBM-EO: *n* = 25), pain was mild to moderate and peaked at D5 (PBM-IO: median 3 [IQR: 0–4.25]; PBM-EO: median 1 [IQR: 0–4]), with no significant differences between groups at D3 (*p* = 0.839), D5 (*p* = 0.531) or D10 (*p* = 0.853) (Table 2). The median pain score at D3 remained 0 in this subgroup, as most lesions developed later in follow-up.

Salivary pH values remained stable and close to neutrality in both groups throughout the follow-up period, with no statistically significant differences between PBM-IO and PBM-EO at any time point. At baseline (D0), median pH values were 7.94 (IQR: 7.64–8.26) and 7.67 (IQR: 7.08–8.34), respectively (*p* = 0.249). This pattern persisted on day 3 (*p* = 0.708), day 5 (*p* = 0.781), and day 10 (*p* = 0.607) (Mann–Whitney *U* test).

## Discussion

This study demonstrated comparable effectiveness of intraoral and extraoral PBM for preventing oral mucositis in pediatric oncology patients undergoing HDMTX. No significant differences were observed in OM occurrence, severity, pain, salivary pH, or plaque index between groups. Multivariable analysis indicated that age and HDMTX dose were more strongly associated with OM severity than PBM modality, supporting the clinical comparability of intraoral and extraoral PBM in this population.

A key finding of this study was the low incidence of oral mucositis in both groups. Overall, 35.5% of patients developed OM, and severe OM (WHO grade 3–4) occurred in only 7.4% of patients evaluated at D10. These rates are markedly lower than those reported in pediatric patients receiving HDMTX without standardized preventive strategies [2, 3]. Valer et al. reported OM in 74.9% of chemotherapy cycles, including ulcerative lesions in nearly half of cases and severe OM in 15.3% [2]. In contrast, the low OM occurrence and limited number of severe cases in the present study reinforce the preventive effect of PBM, consistent with previous studies demonstrating its efficacy in reducing chemotherapy-induced OM in pediatric patients [14, 18, 19]. Importantly, the predominance of mucositis-free patients and the low levels of reported pain further highlight the clinical relevance of preventive PBM in maintaining patient comfort during cancer treatment [18, 20–22].

Exploratory analyses revealed descriptive differences in OM severity between groups at later time points, with fewer advanced lesions and a higher proportion of mucositis-free patients in the PBM-EO group at D5 and D10. However, these differences were not statistically significant and should be interpreted in light of the age imbalance between groups. Despite randomization, patients in the PBM-IO group were older than those in the PBM-EO group (median 8 vs. 6 years), which likely occurred by chance. In the multivariable ordinal regression model, PBM modality was not independently associated with OM severity, whereas increasing age emerged as a significant predictor of more severe mucositis.

Preventing progression to severe OM is clinically relevant, as advanced lesions are associated with increased pain, impaired oral intake, and possible interruptions in cancer treatment. Previous studies on intraoral PBM have consistently demonstrated reductions in severe OM rather than complete prevention of lesion onset [21, 23, 24]. However, direct comparisons with extraoral approaches remain limited, highlighting the novelty of the present study. The comparable performance of both protocols [10, 25, 26] suggests that PBM benefits may involve broader biological effects beyond direct mucosal irradiation, including anti-inflammatory modulation, microcirculatory enhancement, and redox state regulation [13, 27–29], that can also be achieved through extraoral application [30, 31]. Additionally, Monte Carlo simulations demonstrated that near-infrared light applied transcutaneously can reach the oral mucosa with therapeutically relevant fluences [30], and reduced tissue thickness in pediatric patients may further facilitate light penetration.

From a practical perspective, extraoral PBM may facilitate clinical implementation. Previous studies demonstrated that extraoral protocols can be delivered in substantially less time than intraoral irradiation while maintaining comparable efficacy [25], largely due to devices with larger spot sizes that allow broader tissue coverage with fewer application points [10]. In addition, the extraoral approach is non-invasive, improving patient comfort while potentially reducing the risk of cross-contamination [10, 32]. These characteristics also expand its applicability to patients with limited mouth opening, severe mucositis, or low cooperation, including pediatric and critically ill populations [30, 33]. Furthermore, extraoral PBM may also provide broader anatomical coverage, potentially reaching oral and oropharyngeal sites as well as adjacent structures such as salivary glands [30, 34].

Despite the established benefits of PBM, there is substantial variability in treatment parameters, particularly for extraoral protocols. In the present study, a red wavelength (660 nm) was used, which is widely adopted in intraoral applications [29, 35]. However, wavelength selection is especially relevant in extraoral applications, as near-infrared wavelengths may provide deeper tissue penetration and improved energy delivery to the oral mucosa [36]. Additionally, tissue attenuation and scattering make it difficult to estimate the actual dose reaching target tissues during extraoral irradiation [30]. Therefore, further studies are needed to establish optimal wavelengths, irradiation sites, and treatment parameters for EO-PBM, particularly in pediatric populations.

Secondary outcomes, including pain, salivary pH, and plaque index, were analyzed to explore additional clinical effects of PBM beyond OM prevention. Overall, pain levels remained low throughout the follow-up period in both groups, with a high proportion of patients reporting no pain at all time points, which is consistent with the reduced occurrence and severity of OM observed. Even among patients who developed OM, pain was mild to moderate and comparable between groups. These findings reinforce the role of PBM in maintaining patient comfort during chemotherapy [21]. Salivary pH values remained stable and within a near-neutral range at all evaluated time points, suggesting maintenance of oral homeostasis during chemotherapy [37]. Similarly, no significant differences in plaque index were identified between groups, minimizing its potential confounding effect on oral mucositis outcomes. Taken together, these findings support the safety and stability of both PBM approaches.

This study has limitations. It was not prospectively registered and despite randomization, younger patients were more frequently allocated to the extraoral group, an imbalance that likely occurred by chance, as simple randomization was used. Although age was included as a covariate in the multivariable analysis, residual confounding cannot be completely excluded. Moreover, HDMTX dose data were available for only about half of the sample, as this information was not systematically reported during the earlier study period; thus, dose-related findings should be interpreted with caution. Furthermore, pain in very young children was reported by parents or caregivers, which may not accurately reflect the child's experience. Finally, the single-center design and lack of participant/operator blinding may limit generalizability and introduce bias, although outcome assessment was blinded.

## Conclusion

In conclusion, intraoral and extraoral photobiomodulation demonstrated comparable efficacy in the prevention of chemotherapy-induced oral mucositis in pediatric patients undergoing high-dose methotrexate. Both approaches were associated with low rates of severe oral mucositis, minimal pain levels, and stable oral clinical parameters throughout follow-up. Given its non-invasive application, shorter treatment time, and greater practicality in pediatric settings, extraoral PBM may represent a feasible alternative to conventional intraoral protocols. Further multicenter studies with standardized parameters are warranted to confirm these findings and optimize PBM protocols for pediatric oncology care.

## Data Availability

No datasets were generated or analysed during the current study.
